# Ultrasound enhanced the conjugation of epigallocatechin gallate on whey protein isolate and its influence on the emulsifying property and allergenicity

**DOI:** 10.3389/fnut.2025.1604708

**Published:** 2025-07-02

**Authors:** Lilan Chen, Haohui Li, Chunyuan Jiang, Baoer Zuo, Meifeng Li, Sining Li, Xiaoning Zhang

**Affiliations:** ^1^Sichuan Cuisine Development and Research Center, Sichuan Tourism University, Chengdu, China; ^2^School of Public Health, Chengdu University of Traditional Chinese Medicine, Chengdu, China; ^3^School of Food Science & Engineering, Qilu University of Technology (Shandong Academy of Science), Jinan, China; ^4^College of Food Science and Technology, Southwest Minzu University, Chengdu, China

**Keywords:** whey protein isolate (WPI), epigallocatechin gallate (EGCG), ultrasound, allergenicity, emulsifying property

## Abstract

**Background:**

The conjugation of polyphenols to proteins provides a method for modifying the structure and properties of proteins.

**Methods:**

This study investigated the roles of ultrasound in the conjugation of epigallocatechin gallate (EGCG) with whey protein isolate (WPI) and its effects on the structural characteristics and properties.

**Results and discussion:**

The formation of EGCG-WPI conjugates (EW) resulted in a decrease in free amino groups and thiol groups in WPI, accompanied by an increase in size and thermal stability. Consequently, this conjugation inhibited the immunoglobulin E (IgE) binding capacity and improved the emulsifying properties of WPI. Furthermore, ultrasound facilitated the interaction by producing larger size of conjugates (U-EW), increasing the binding affinity from 5.8 × 10^5^ M^−1^ to 1.7 × 10^6^ M^−1^ and the polyphenol bound equivalent from 80.4 ± 1.3 mg/g to 98.2 ± 1.9 mg/g compared to EW. It induced the greater changes in the secondary structure and surface hydrophobicity, thereby promoting greater participation of β-lactoglobulin (βLg) in conjugation with EGCG, and resulting in a higher inhibition rate of IgE binding capacity, an enhanced emulsifying property of U-EW. These findings will potentially expand the applications of WPI in the food industry.

## 1 Introduction

Whey protein isolate (WPI), consists of β-Lactoglobulin (βLg), α-lactalbumin (αLa), bovine serum albumin (BSA) and lactoferrin (LF), offers an exceptionally high nutritional value with all the essential amino acids required by humans (1, 2). It is well known that WPI is extensively utilized as ingredient in dairy products, meat products, and baked goods to enhance the nutritional value of these products, as well as to improve their texture, taste, and stability (3, 4). However, its emulsifying properties vary under different environmental conditions, which is the key functional property that governs its application in dairy and meat products. Furthermore, it has potential allergenic effects that could trigger milk allergies, classified as immunoglobulin E (IgE)-mediated type I hypersensitivity (5, 6). This can lead to a severe inflammatory response and various allergic symptoms, potentially resulting in life-threatening reactions (6).

The emulsifying properties and allergenicity of WPI are closely dependent on its conformational structure (7). Consequently, these properties can be modified or inhibited by altering the conformational structure of WPI. Chemical conjugation offers a method to alter the structure through either covalent or non-covalent bonds. Interestingly, polyphenols, which contain multiple phenolic hydroxyl groups, exhibit a significant potential for combining with WPI to form polyphenol-WPI conjugates (8, 9). The formation of these conjugates primarily relies on hydrogen bonds, hydrophobic interactions, electrostatic interactions, and covalent bonding (10). Moreover, this conjugation can modify the secondary structure of WPI, thereby influencing its properties, such as enhancing emulsifying ability, reducing allergenicity and masking unpleasant odors (10, 11).

Stirring and shaking at alkaline condition is the commonly used method for the conjugation of WPI and polyphenols. However, it faces challenges, including low binding efficiency, prolonged conjugation time, and harsh conditions (12). Fortunately, ultrasonic technology has emerged as a green and efficient physical processing method, gaining widespread application in the food industry (6, 13). Ultrasound is a mechanical wave with a frequency exceeding 20 kHz, which generates a cavitation effect (13). These effects can disrupt chemical bonds between molecules, enhance molecular interactions, and accelerate the rate of chemical reactions (14). For instance, our previous studies revealed that ultrasound facilitates the adsorption of β-lactoglobulin on starch nanoparticles, thereby enhancing desensitization effects (6). Chinarak et al. (15) demonstrated that ultrasonic treatment significantly improved the interaction of WPI and gallic acid, promoting structural changes. However, the mechanisms by which ultrasound affects the conjugation of polyphenols to WPI and its influence on properties, particularly emulsifying ability and allergenicity, remain unclear.

In this study, the effect of ultrasound on the conjugation of WPI and epigallocatechin gallate (EGCG) was investigated from the perspectives of binding affinities (Ka), secondary structure, and protein compositions of WPI-EGCG conjugates. Furthermore, the resulting alteration in allergenicity and emulsifying properties were probed. These results will provide a framework for optimizing the characteristics of WPI and expanding the application of ultrasound in the food industry.

## 2 Materials and methods

### 2.1 Materials

Whey protein isolate (protein content 93.5%) was purchased from Mullins Whey Co., Ltd. (Mosinee, WI, United States). The epigallocatechin gallate (purity ≥ 99%), 1-anilinonaphthalene 8 sulfonate (ANS) and ortho-phthaldialdehyde (OPA) were purchased from Sigma Aldrich (St. Louis, MO, United States). Serum samples from cow milk allergen (CAM) patients were generously provided by Jinan Maternity and Child Care Hospital (Jinan, China). Other chemical reagents, including PBS, NaOH and Folin–Ciocalteu are analytical grade and purchased from Sinopharm Chemical Reagents Co., Ltd. (Shanghai, China).

### 2.2 Formation of EGCG-WPI conjugates

The EGCG-WPI conjugates were formed following the protocols established by Shao et al. with minor modifications (9). WPI (2 mM, 0.1 mM) was incubated with EGCG solution (2 mL, 1 mM) in alkaline environment (pH 9.0) at 24°C for 2 h. Afterwards, EGCG-WPI conjugates, referred to EW, were obtained after removing unreacted EGCG by dialysis (2000 Da cut-off) against double-distilled water at 4°C with 4 water changes. For the ultrasound treatment (U-EW), these solutions were processed using an ultrasound probe with a circulating water bath at 24°C (JY92-IIDN, Ningbo Scientz Bio-technology Co., Ningbo, Zhejiang, China). The sonication was set to 120 W, with a pulse duration of on-time 4 s and off-time 2 s according to previous studies.

### 2.3 Polyphenol bound equivalents

The phenol contents in EW and U-EW were measured using the Folin–Ciocalteu method (16). Briefly, EGCG (0.5 mg/mL) was utilized as the standard solution to construct the calibration curve. The resulting standard equation is *y* = 9.16452*x* + 0.00642, with an *R*^2^ value of 0.9995. One milliliter of each sample was mixed with Folin–Ciocalteu (2.5 mL, 0.1 N) for 5 min, then co-incubated with 2.5 mL sodium carbonate (15%, w/v) at 40°C for 1 h. The absorbance at 778 nm was determined using an ultraviolet–visible spectrophotometer, with the protein sample treated in the same manner as a control.

### 2.4 Free amino groups and thiol groups

The content of free amino groups in WPI and EGCG-WPI conjugates was determined using ortho-phthaldialdehyde (OPA) method (17). Two hundred microliters of samples (4 mg/mL) was incubated with 4 mL of the OPA reagent at 35°C for 2 min. The absorbance at 340 nm was measured against the OPA reagent. The content of free thiol groups was measured according to the method described by (38). Fifteen milligrams of samples were incubated with 50 μL of Ellman reagent at room temperature for 1 h. The absorbance at 412 nm was measured, using the conjugates as a blank.

### 2.5 Dynamic light scattering (DLS)

WPI and EGCG-WPI conjugates solution were filtered through a PTFE 0.8 μm filter, added in Malvern ZetaSizer Nano ZS instrument to measure the hydrodynamics diameters and zeta-potential with refractive index of 1.450 according previous study (3).

### 2.6 Fluorescence spectroscopy

The fluorescence intensity of WPI and EGCG-WPI conjugates measured on a Cary Eclipse fluorescence spectrophotometer (Varian, Sweden). WPI (0.1 mM) was incubated with EGCG (0–1 mM) at 24°C for 30 min in the presence or absence of ultrasound, respectively. These samples were excited at 280 nm with a slit width of 5 nm and the emission spectra was recorded from 300 to 450 nm. The binding affinities was calculated following Equation:


(1) $$\log[(F_{0}-F)/F]=\log\;\mathrm{Ka}+n\;\log[D]$$

Where F_0_ and F are the maximum fluorescence intensities of WPI in the absence and presence of the EGCG, respectively, D is the EGCG concentration, and *n* is the number of binding sites.

### 2.7 Circular dichroism (CD)

The secondary structure of WPI in native and in conjugates were determined by a Chirascan Spectropolarimeter (Applied Photophysics, United Kingdom) operating at 200 nm/min speed, 2.0 nm bandwidth with a 1 mm path length quartz cell at 24°C.

### 2.8 Surface hydrophobicity (H_0_)

The H_0_ of WPI and EGCG-WPI conjugates was determined using 8-aniline-1-naphthalene sulfonate (ANS) as the fluorescent probe as described by Han et al. (4).

### 2.9 Differential scanning calorimetry (DSC)

The thermal stabilities of WPI and EGCG-WPI conjugates were determined using a DSC 8000 thermal analysis system (Shimadzu, Tokyo). Briefly, 5.0 mg of sample powder was heated from 50°C to 200°C with a constant rate of 5°C/min under dry nitrogen.

### 2.10 Sodium dodecyl sulfate polyacrylamide gel electrophoresis (SDS-PAGE)

SDS-PAGE analysis was performed following our previous protocols with slight modification (18). The precast PAGE gels utilized were 5% stacking gels and 15% separating gels. The conjugates were mixed with loading buffer and boiled for 5 min before electrophoresis. A volume of 10 μL from each sample solution was cooled to room temperature and then loaded into the gel lanes, with a working voltage set at 180 V.

### 2.11 IgE binding capacity

The IgE binding capacity of WPI and EGCG-WPI conjugates were determined by an inhibition enzyme-linked immunosorbent assay (ELISA) described by Zhang (19). The polystyrene MaxiSorp 96 U-well microplates (Roskilde, Denmark) were coated by 120 μL WPI and EGCG-WPI conjugates (5 μg/mL), then incubated with 100 μL CAM patients’ serum (1:30 in PBST) for 2 h after the free-binding sites were blocked. Afterwards, goat anti-human IgE-HRP (100 μL, 1:200 in PBST) was added and incubated for 60 min. The absorbance of microplates at 450 nm was determined by a Bio-Rad Microplate Reader. The inhibition rate was calculated using:


(2) $$\text{Inhibition}\;(\%)=(1-B/B_{0})\times 100$$

where B and B_0_ are the absorbance values of the wells in the WPI or EGCG-WPI conjugates, respectively.

### 2.12 Emulsifying ability (EAI) and emulsification stability (ESI)

The emulsifying properties of WPI before and after EGCG conjugates was access according to our protocols (20). One milliliter of soybean oil was added to 9 mL of sample solution (1 mg/mL) and homogenized at 10,000 rpm for 2 min. Subsequently, 50 μL of the emulsion was taken and incubated with 5 mL of 9% SDS. The absorbance of emulsions at 500 nm was determined. The EAI and ESI was calculated by the following Equations:


(3) $$\mathrm{EAI}\;(m^{2}/g)=\frac{2\times 2.303}{c\times \emptyset \times L\times 10^{4}}\times A_{0}\times D$$

(4) $$\mathrm{ESI}\;(\min)=\frac{A_{0}}{A_{0}-A_{10}}\times 10\;$$

Where c is the concentration of emulsion, A_0_ and A_10_ are the absorbance of emulsions at 0 min and 10 min, *Φ* is the oil phase volume percent, D is the dilution factor (100), L is the light range (2.303).

### 2.13 Statistical analysis

The measurements were performed in triplicate, and the data are presented as mean ± standard deviation. Statistical analysis was conducted using SPSS version 17.0 (SPSS Inc., Chicago, IL, United States). A significant difference was defined as *p* < 0.05 and represented by different letters.

## 3 Results and discussion

### 3.1 The polyphenol bound equivalents

The alkaline conditions can oxidize EGCG to quinones, further extend the structure to maintain the deprotonation of WPI, facilitating the conjugation of EGCG to WPI (4). The polyphenol binding equivalents of EGCG-WPI conjugates, both with and without ultrasound treatment, were analyzed using the Folin-Phenol assay. The results indicated that the equivalents in the EW and U-EW were 80.4 ± 1.3 mg/g and 98.2 ± 1.9 mg/g, respectively. This increase in bound equivalents demonstrates that ultrasound can enhance the conjugation of EGCG to WPI, induced by the cavitation and shearing effects of ultrasound (6).

### 3.2 Free amino group and thiol group

**Figure 1:**
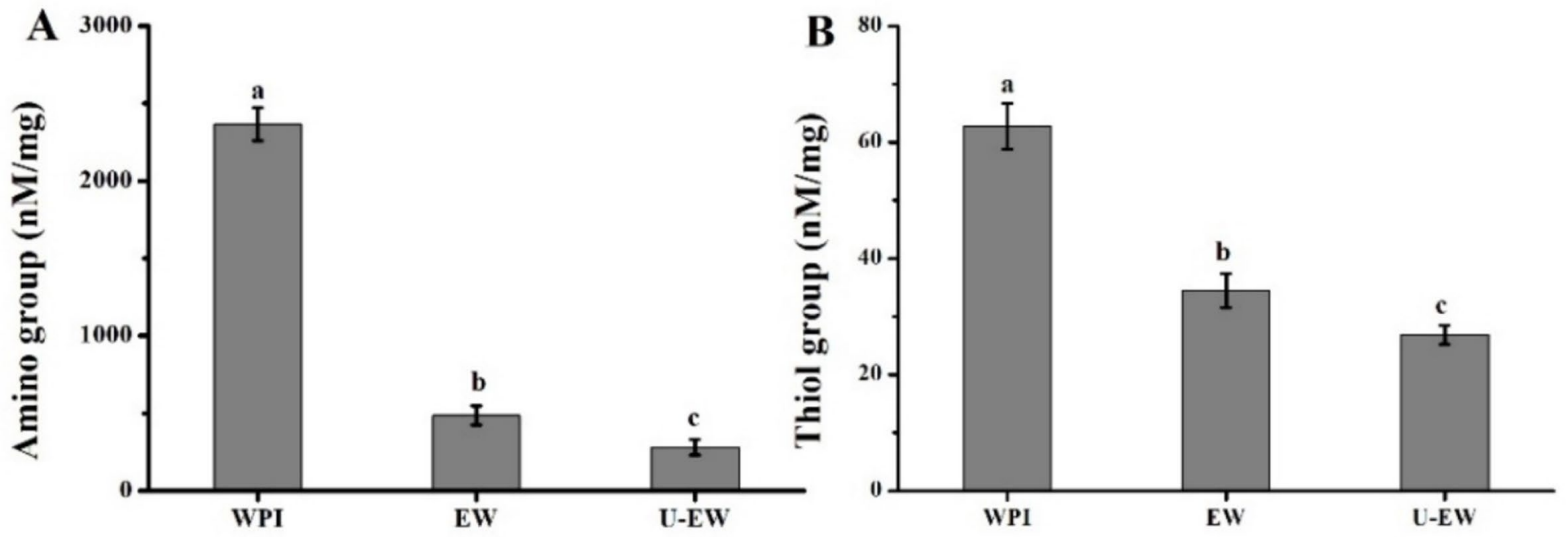
The content of free amino groups **(A)**, thiol groups **(B)** in WPI, EW and U-EW. Different letters represent significant differences (*p* < 0.05) and error bars correspond to standard errors.

The quinones derived from phenolic compounds are susceptible to react with the nucleophilic side chains of proteins, such as amino and thiol groups (21). The conjugation of EGCG with WPI was further investigated by monitoring changes in the concentration of free amino and thiol groups, and showed in Figure 1. The conjugation of EGCG with WPI significantly decreased the levels of free amino groups, as the result of the free radicals combining with polyphenols. Furthermore, ultrasound treatment resulted in a more significant reduction of free amino groups in the conjugates (U-EW), with EW showing 500 ± 70 nM/mg, while U-EW demonstrated 300 ± 50 nM/mg. Additionally, the alteration in thiol groups exhibited a similar trend with changes in amino groups. The thiol group concentration decreased from 62.7 ± 3.9 nM/mg in WPI to 34.4 ± 2.9 nM/mg in EW, and further to 26.8 ± 1.6 nM/mg in U-EW.

### 3.3 DLS

**Figure 2:**
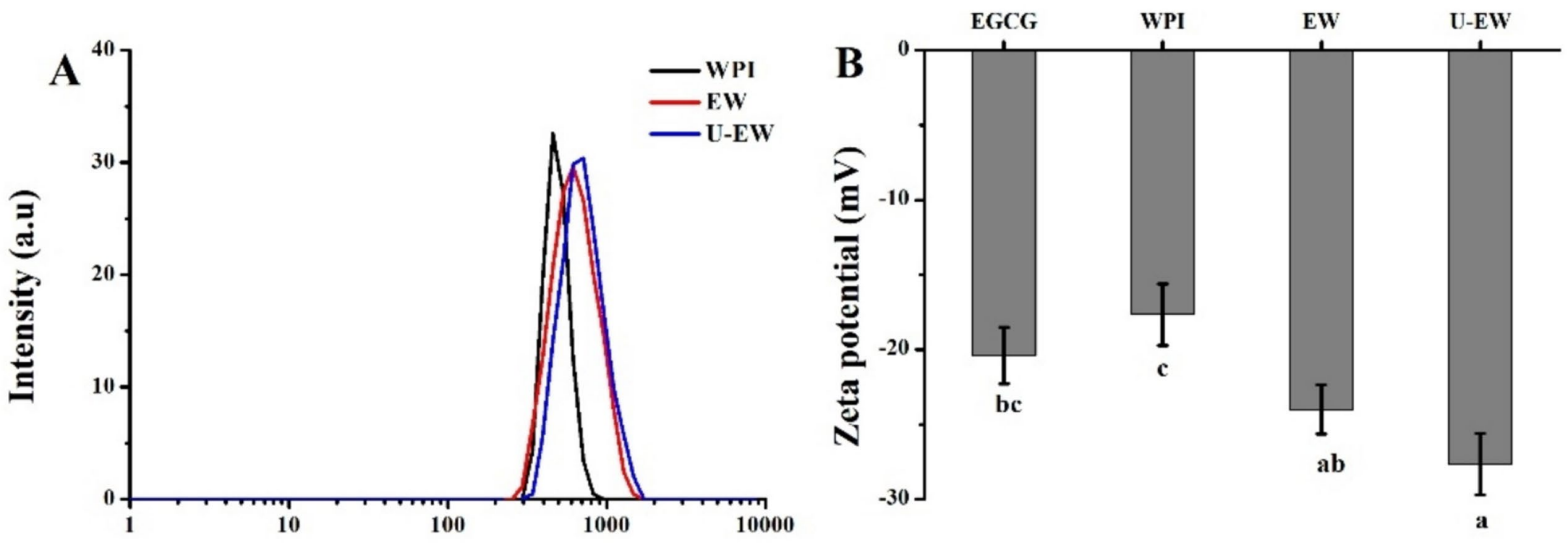
The DLS spectra **(A)** and zeta potential **(B)** of WPI, EW and U-EW. Different letters represent significant differences (*p* < 0.05).

The DLS is a widely used method for determining the particle size distribution based on Brownian motion in liquids (22), and also employed to assess the degree of conjugation of polyphenols with proteins (23). The hydrodynamic diameters and zeta potentials of WPI before and after EGCG conjugation are illustrated in Figure 2. The hydrodynamic diameter increased from 450 ± 44 nm for WPI to 600 ± 59 nm in EW. The zeta potential of EW was approximately −24.1 ± 1.6 mV, which is lower than that of WPI (−17.7 ± 2.1 mV). Zeta potential is a critical indicator of emulsion stability, reflecting the extent of electrostatic repulsion between droplets (24). The increase in the absolute value of the zeta potential indicates that the stability of WPI was enhanced by the conjugation of EGCG, moreover, the negative charge of EGCG could enhance the negativity of the conjugates. Additionally, the diameter of the U-EW increased to 710 ± 52 nm, which is larger than that of EW, and it exhibited the highest absolute zeta potential value of 27.9 ± 2.0 mV. The sonication process facilitated the conjugation of EGCG, resulting in a reduction of interfacial tension and creating a robust barrier against flocculation and coalescence (25).

### 3.4 Fluorescence spectroscopy

The WPI contains plenty of chromophores, namely, tryptophan, tyrosine, and phenylalanine residues, which provide a convenient parameter for investigating the interactions between proteins and ligands (26). The fluorescence intensity can be quenched when the chromophores are within a quenching distance to EGCG, inducing fluorescence resonance energy transfer and resulting in fluorescence quenching (22). Furthermore, the quenching efficiency depends on the binding force and distance, which can be described by the Ka (6).

**Figure 3:**
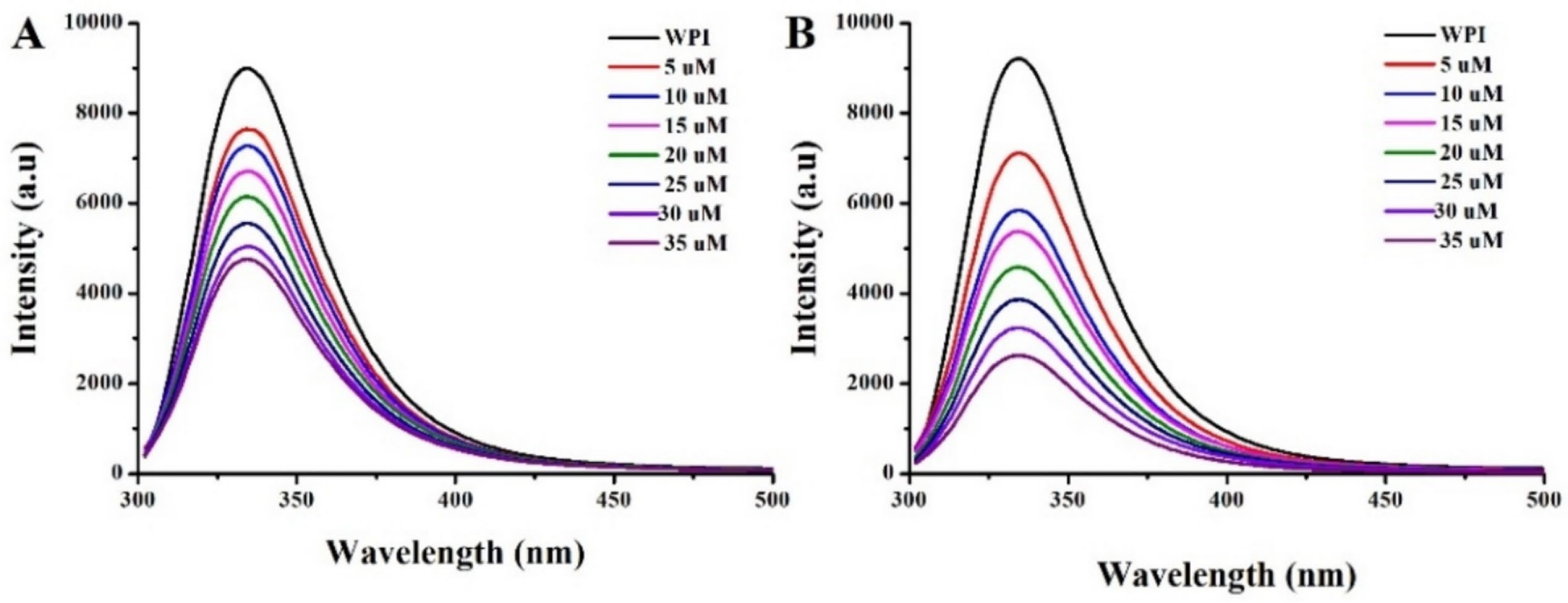
Fluorescence emission spectra of WPI after incubation with EGCG without **(A)** and with **(B)** ultrasound treatment.

The fluorescence spectrum of WPI before and after EGCG conjugated with or without ultrasound treatment, are illustrated in Figure 3. A redshift in the maximum emission wavelength (λmax) was observed when WPI was conjugated with EGCG, which was further enhanced by ultrasound treatment. This result indicates the unfolding of the protein, leading to conformational changes that expose more tryptophan residues to a more hydrophilic environment (27). Furthermore, the fluorescence intensity of WPI gradually decreased with the increasing concentration of EGCG, indicating the occurrence of intermolecular energy transfer between WPI and EGCG. Notably, sonication accelerated this quenching rate, resulting in an increase in Ka from 5.8 × 10^5^ M^−1^ to 1.7 × 10^6^ M^−1^ when the conjugation occurred in an ultrasound environment accroding to Equation 1. This result aligns with the findings of Shao, who reported that ultrasound increased the Ka of EGCG with βLg from 2.5 × 10^5^ M^−1^ to 6.3 × 10^5^ M^−1^ (9). This phenomenon can be attributed to two factors (6): first, the cavitation effect generated by ultrasound facilitates the conjugation of EGCG to WPI; second, ultrasound promotes the unfolding of the protein (3), thereby increasing the exposure of active sites in WPI to EGCG. Consequently, the number of binding sites (n) increased from 1.04 to 1.56.

### 3.5 Secondary structure and H_0_

The changes in the secondary structure of WPI before and after conjugation with EGCG were analyzed using CD, as shown in Table 1. The secondary structure of WPI consists of 12% *α*-helix, 39% *β*-sheet, 17% β-turn, and 32% random coils. However, upon conjugation with EGCG, the proportion of α-helix increased while the β-sheet proportion decreased. Additionally, ultrasound treatment enhanced this alteration, consistent with the findings of Ka. These results suggest that conjugation may lead to a partial expansion of the WPI molecular structure and induce the conversion of β-sheet to α-helix. The reduction of β-sheet, particularly βLg, could disrupt the conformation of IgE epitopes, emphasizing the potential for desensitization to WPI (28).

**Table 1:** Secondary structural components of WPI in native and conjugates states as affected by ultrasound treatment.

Samples	α-helix	β-sheet	β-turn	Random coil
WPI	12%	39%	17%	32%
EW	16%	33%	20%	31%
U-EW	18%	30%	16%	36%

**Figure 4:**
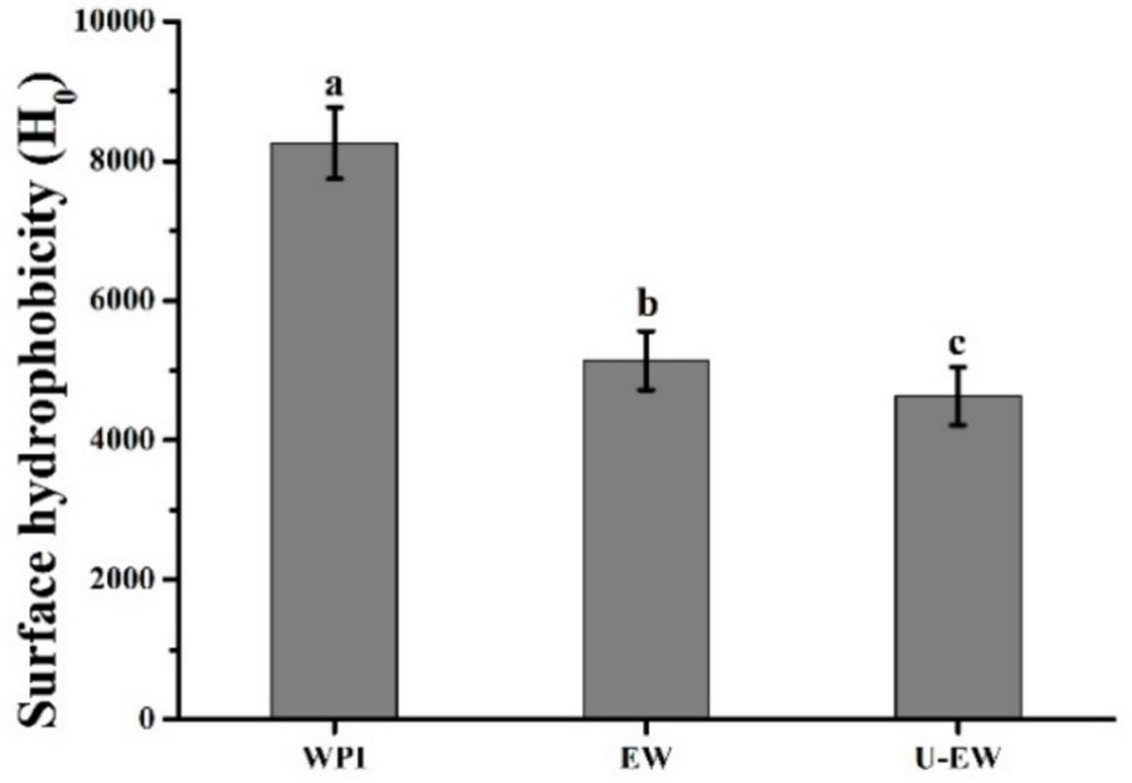
Surface hydrophobicity of WPI, EW and U-EW. Different letters represent significant differences (p < 0.05).

The H_0_ is another structural characteristic of proteins that influences their properties, specifically related to the number of hydrophobic groups present on the protein surface (29). As shown in Figure 4, the intensity of H_0_ decreased from 8,200 ± 520 in WPI to 5,100 ± 460 in EW, and further decreased to 4,600 ± 480 in U-EW. The conjugation of EGCG induced protein unfolding and resulted in a more hydrophilic profile compared to WPI, exposing hydrophobic amino acid residues that were previously buried within the globular structure of the protein. It has been established that hydrophobic interactions drive the molecular rearrangement, which is facilitated by ultrasound, leading to the formation of conjugates (30).

### 3.6 DSC

**Figure 5:**
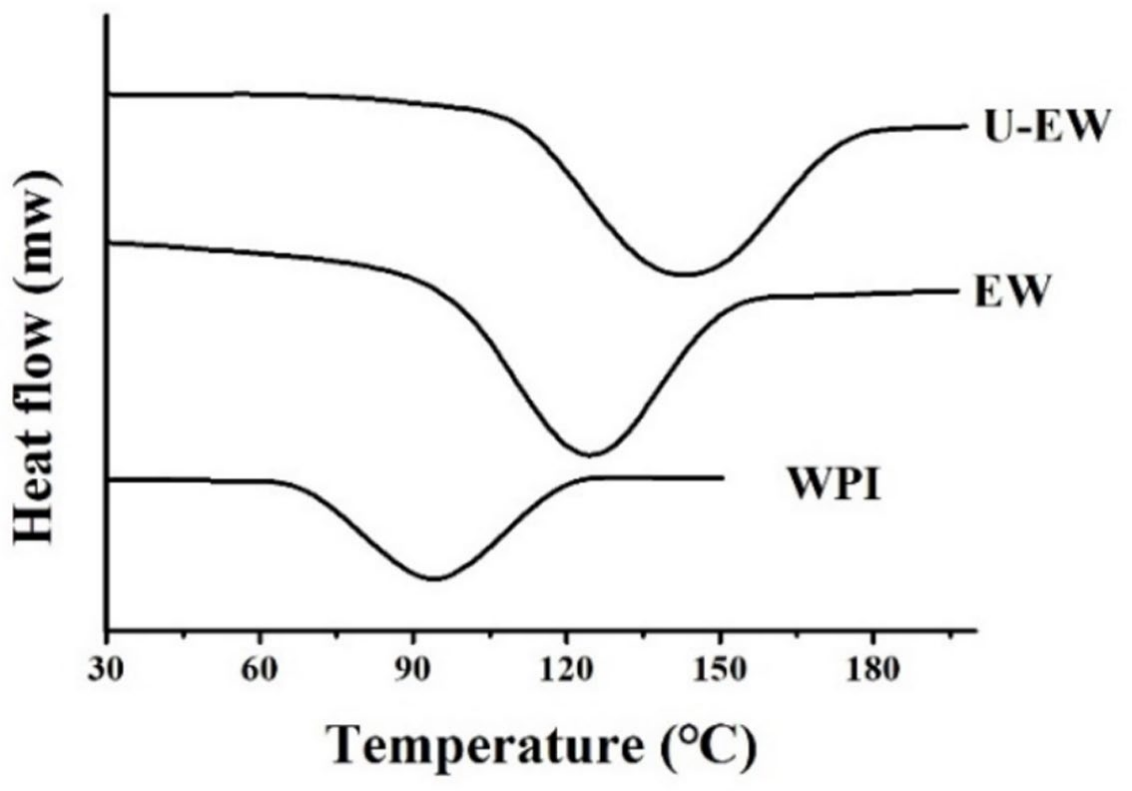
DSC analysis of WPI, EW and U-EW.

The DSC is utilized to characterize the denaturation temperature of the conjugates by measuring heat flow during temperature changes, as presented in Figure 5. The results indicate that the denaturation temperatures of the conjugates were significantly increased compared to native WPI. WPI exhibited a characteristic peak at 94.53 ± 0.62°C with a ΔH of 351.27 ± 55.28 J/g, while the characteristic peak of EW increased to 129.64 ± 0.53°C, and further rose to 142.42 ± 0.86°C in the U-EW. This demonstrates that the attachment of EGCG to the protein molecules enhances the thermal stability of the conjugates. The higher denaturation temperature suggests the improved thermal stability (6). The conjugation of EGCG may reduce peptide degradation, and furthermore, the crosslinking decreases the content of free amino acids, thereby enhancing thermal stability (31).

### 3.7 SDS-PAGE and IgE binding capacity

β-lactoglobulin is the primary allergenic protein found in WPI, with a molecular weight of 18.4 kDa (32). Consequently, desensitization to βLg is the primary strategy for reducing WPI allergy, and the IgE binding capacity is a key criterion for assessing the allergy (33, 34).

**Figure 6:**
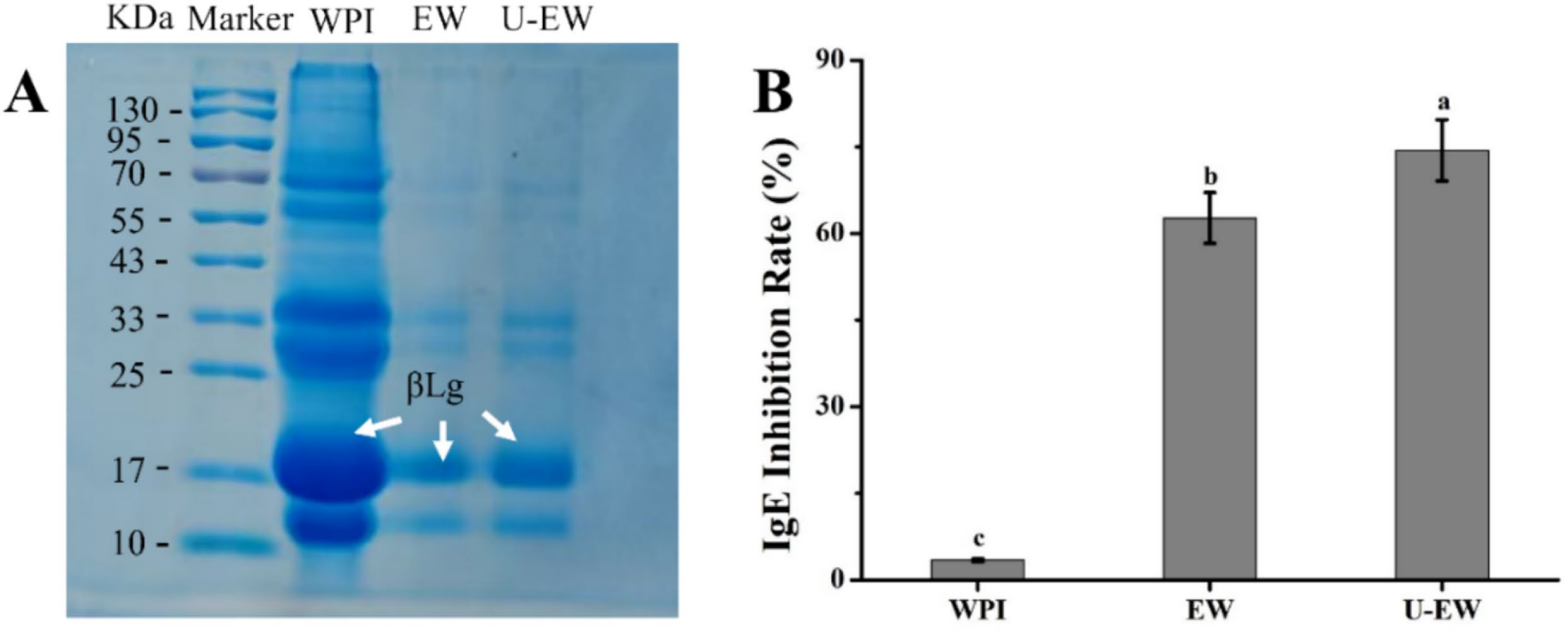
SDS-PAGE **(A)** and IgE inhibition rate **(B)** of WPI, EW and U-EW. Different letters represent significant differences (*p* < 0.05).

SDS-PAGE under denaturing conditions was employed to analyze the changes in molecular weights changes in WPI before and after EGCG conjugation. Figure 6A presents the electrophoretic results for WPI, EW, and U-EW, respectively. Compared to the marker, the native WPI exhibited two prominent bands at approximately 18 kDa and 14 kDa, corresponding to βLg and αLa, respectively. However, the bands for EW and U-EW migrated slightly upward compared to native WPI, indicating successful EGCG conjugation. Notably, observing the electrophoretic spectra of EW and U-EW, it can be found that the bands at 18 kDa in U-EW appeared more denser than that of EW, suggesting that may promote greater participation of βLg in conjugation with EGCG, leading to a higher relative representation of βLg-related conjugates (33).

The effect of WPI-EGCG conjugates on the IgE inhibition rate was determined using ELISA, as illustrated in Figure 6B. The IgE binding capacity of WPI was significantly reduced when conjugates were formed (>60%, *p* < 0.05) accroding to Equation 2. Furthermore, the IgE combing inhibition rate of U-EW increased to 74.4% ± 5.3% due to the enhanced conjugation of EGCG with WPI facilitated by ultrasound. The conjugation of EGCG demonstrated a greater desensitization effect compared to the proteolytic hydrolysis of WPI, which had an IgE binding capacity of approximately 61.65% (35). Additionally, the SDS-PAGE results indicated that more βLg was involved in the conjugation process under ultrasound, leading to a more effective inhibition of allergies, as βLg is the primary allergenic protein in WPI.

### 3.8 EAI and ESI

**Figure 7:**
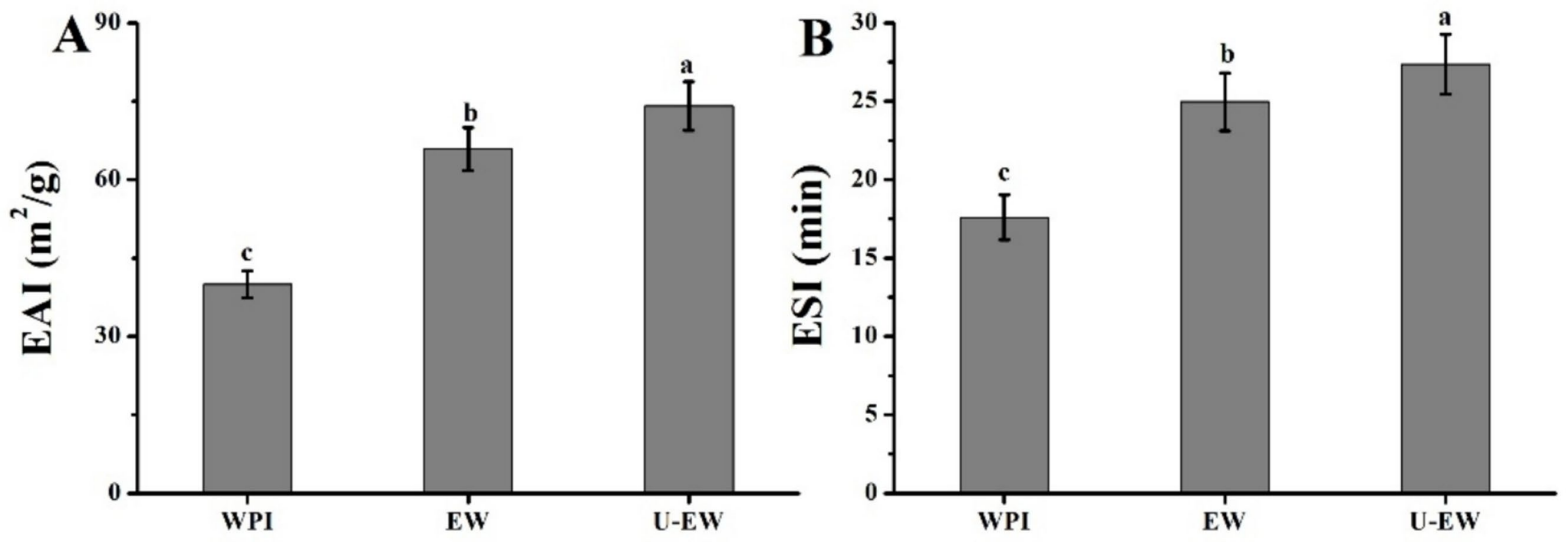
Emulsifying properties of WPI, EW and U-EW. **(A)** EAI and **(B)** ESI. Different letters represent significant differences (*p* < 0.05).

The EAI and ESI are the key criteria for evaluating the functional properties of proteins and their applications (11). Enhanced EAI and ESI can broaden their use in the food industry (36). The EAI and ESI of WPI in native state, as well as in EGCG-WPI conjugates formed with and without ultrasound treatment, were assessed in soybean oil emulsions, with the results calculated by Equations 3 and 4 presented in Figure 7. The findings indicate that ultrasound treatment can enhance the conjugation of EGCG to WPI, thereby facilitating an increase of ESI and EAI in U-EW. The EAI of WPI significantly increased from 41.2 ± 2.9 m^2^/g to 65.9 ± 4.1 m^2^/g after conjugation with EGCG, and further increased to 74.1 ± 4.7 m^2^/g following ultrasound treatment. Similarly, the ESI of native WPI was measured at 17.6 ± 1.4 min, which increased to 24.9 ± 1.8 min in emulsions without ultrasound (EW) and to 27.7 ± 1.7 min in U-EW. The covalent conjugation of EGCG enhances the long-range steric repulsion of WPI, forming a stable membrane around the oil droplets, which contributes to greater emulsion stability. The findings were consistent with the research conducted by Meng and Li (12), which reported that the conjugation of WPI and polyphenols through non-covalent interactions could enhance both EAI and ESI more effectively than WPI alone. Furthermore, the presence of aromatic residues may also improve the emulsion characteristics of WPI (37).

## 4 Conclusion

The conjugation of EGCG increased the hydrodynamic diameters of EW and the absolute value of the zeta potential of WPI, while significantly decreasing their amino groups and thiol groups from 2,370 ± 110 nM/mg to 490 ± 40 nM/mg, and from 62.7 ± 3.9 nM/mg to 34.4 ± 2.9 nM/mg, respectively. Consequently, the secondary structures of WPI were changed, leading to a reduction in IgE binding capacity and an improvement in emulsifying properties. Furthermore, ultrasound facilitated this conjugation by forming larger conjugates (U-EW), which increased the Ka from 5.8 × 10^5^ M^−1^ to 1.7 × 10^6^ M^−1^, and the polyphenol-bound equivalent increased from 80.4 ± 1.3 mg/g to 98.2 ± 1.9 mg/g. This resulted in significantly changes in the secondary structure and surface hydrophobicity decreased from 8,200 ± 520 in WPI to 5,100 ± 460 in EW, and further decreased to 4,600 ± 480 in U-EW. Additionally, the effects of ultrasound promoted greater involvement of βLg to EGCG when conjugates formation, leading to a higher inhibition rate of IgE binding capacity and enhanced emulsifying properties. However, some limitations, such as the process conditions for large-scale applications, the effects of modifications, and the influencing factors, still require comprehensive research based on this finding. Therefore, future research work should consider a more detailed analysis of the influencing factors and modification effects. These findings highlight the potential applications of WPI in the food industry.

## Data Availability

The original contributions presented in the study are included in the article/supplementary material, further inquiries can be directed to the corresponding authors.
